# Comparison of burnout pattern between hospital physicians and family physicians working in Suez Canal University Hospitals

**DOI:** 10.11604/pamj.2014.18.164.3355

**Published:** 2014-06-19

**Authors:** Amany Ali Kotb, Khalid Abd-Elmoez Mohamed, Mohammed Hbany Kamel, Mosleh Abdul Rahman Ismail, Abdulmajeed Ahmed Abdulmajeed

**Affiliations:** 1 Faculty of Medicine- Suez Canal University, Ismailia, Egypt

**Keywords:** Burnout, risk factors, family physicians

## Abstract

**Introduction:**

The burnout syndrome is characterized by emotional exhaustion, depersonalization, and low personal accomplishment. It is associated with impaired job performance.

**Methods:**

This descriptive study examined 171 physicians for the presence of burnout and its related risk factors. The evaluation of burnout was through Maslach Burnout Inventory (MBI). The participant was considered to meet the study criteria for burnout if he or she got a “high“ score on at least 2 of the three dimensions of MBI.

**Results:**

In the current study, the prevalence of burnout in hospital physicians (53.9%) was significantly higher than family physicians (41.94%) with (p=0.001). Participants who work in the internal medicine department scored the highest prevalence (69.64%) followed by Surgeons (56.50%) and Emergency doctors (39.39%). On the other hand, Pediatricians got the lowest prevalence (18.75%). Working in the teaching hospital and being married are strong predictors for occurrence of burnout.

**Conclusion:**

There is a significant difference of burnout between hospital physicians and family physicians among the study subjects. Working in the teaching hospital and being married are strong predictors for occurrence of burnout.

## Introduction

The burnout syndrome is characterized by loosing enthusiasm for work (emotional exhaustion), treating people as if they were objects (depersonalization), and having a sense that work is no longer meaningful (low personal accomplishment) and it refers to a negative consequence of chronic work stress[1]. Burnout as a syndrome is present in many individuals under constant pressure. Physicians in particular are frequently overloaded with the demands of caring of sick patients [2]. Previous studies showed that primary care physician reported alarming levels of professional and personal distress as up to 60% of practicing physicians reported symptoms of burnout [3].

Burnout appears to be quite prevalent in both developing and developed countries and probably represents considerable economic, social and psychological costs to employees and employers in these countries. It is remarkably stable when studied across time on the same individuals and this chronic nature of burnout is probably not due to its genetic or personality origins but rather to work-related characteristics [4]. Several studies were conducted to study burnout in the Eastern Mediterranean region; In Yemen it was (11.7%) [5], While among Tunisian primary care doctors there was (33%) suffering from burnout (35%) of them had high score of emotional exhaustion, (21%) had high depersonalization and (40%) had a low score of personal accomplishment [6], and in Saudi Arabia,Selaihem found Prevalence of burnout amongst physicians working in primary care in Riyadh military hospital, Saudi Arabia (2013) was (53.5%) of respondents scored high for EE burnout, (38.9%) for DP and (28.5%) for PA, with (2.78%) scoring high burnout in all three dimensions [7]

It is documented that physician burnout has been associated with impaired job performance, poor health and lead to physician error, these errors can in turn contribute to burnout. Dissatisfaction and distress have significant costs not only for physicians and their families but for patients and health care organizations as well [2]. Physical symptoms of burnout may take many different forms, including insomnia, appetite changes, fatigue, colds or flu, headaches, and gastrointestinal distress. Physical symptoms alone may interfere with one´s sense of well-being and ability to function fully at work [8], Psychological symptoms such as low or irritable mood, cynicism, and decreased concentration can negatively affect productivity and rapport [9]

Additional components of burnout may include daydreaming while with patients, excessive cancellations, procrastination, and delaying paperwork and vocational tasks [10]. Burnout may also lead to increased alcohol or drug use, which can also impact patient care [11]. The consequences of burnout among practicing physicians include not only poorer quality of life and lower quality of patient care but also a decline in the stability of the physician workforce. It is reported that there has been a major decrease in the percentage of graduates entering careers in primary care in the last 20 years, with reasons related to burnout and poor quality of life [12], this trend, coupled with attrition among currently practicing physicians have a significant effect on patient access to primary care services [13]. On the other side, there is evidence that the well-being of physicians is related to patient satisfaction, a key outcome variable tracked by most organizations.

The satisfaction of physicians in an organization will enhance recruitment and retention of staff, saving the enormous cost of staff and physician turnover [14] Physician well-being prevents burnout and the less frequent but significant problem of physician impairment [15]. Furthermore, attention to well-being promotes patient safety and reduces the probability of errors thereby diminishing the threat of malpractice litigation [16]

## Methods

This is a descriptive cross-sectional study conducted on physicians working in Suez Canal University Hospital (SCUH) in Ismailia and family practice centers affiliated to Faculty of Medicine-Suez Canal University (FOM/SCU).

**Sampling:** a comprehensive sample of family physicians was included in the study (31). As for physicians working in the other clinical departments within (SCHU), the sample was estimated to be 140 physicians. A stratified random sample was then conducted to include physicians from four major categories: 56 Internal medicine physicians (25residents and 31 assistant lecturers), 40 Surgeons (18 residents and 22 assistant lecturers), 16 Pediatricians (10 residents and 6 assistant lecturers), 28 physicians from Emergency and anesthesiology departments (17 residents and 11 assistant lecturers)

**Procedural process:** selected physicians were invited to participate in the study through a series of mailed and electronic communications in addition to personal interviews with follow-up telephone calls from the researcher.

All participants were then interviewed using a semi-structured questionnaire which included: (1) Demographic data and some work characteristics including (age, gender, marital status, number of years in practice, educational qualification, history of smoking, presence of financial or social problems, suffering from chronic diseases, number of patients per day, practice type most of time, type of employment and hours of work per week within the University Hospitals); (2) Measuring Burnout by using Maslach Burnout Inventory (MBI) which is a self-administered, twenty two item questionnaire that was developed to measure burnout in human services workers and is regarded to be the “gold standard” in measuring burnout. The MBI items are rated on a scale from 0 to 6. It is designed to assess the 3 primary dimensions of burnout: losing enthusiasm for work (emotional exhaustion), having a sense that work is no longer meaningful (low personal accomplishment) and (Depersonalization)[12]). Burnout dimensions were categorized in Table 1 and Table 2. A participant was considered to meet the study criteria for burnout if he or she got a “high” score on at least 2 of the three dimensions of MBI [12].

**Table 1:** Categorization of burnout dimensions

Level of burnout	Emotional exhaustion	Depersonalization	Low Personal accomplishment
**Low**	≤ 16	≤ 6	≤ 31
**Moderate**	17-26	7-12	32-38
**High**	≥27	≥ 13	≥ 39

**Table 2:** Comparison of burnout distribution among hospital and family physicians

Physicianspecialty	Burnout					Significance tes (p-value)
	NO		YES		Total	
	No.	%	No.	%		
**Family Physicians**	18	58.06	13	41.94	31	X^2^=17.63 (0.001)*
**Hospital Physicians**	64	45.71	76	53.90	140	

Statistically significant at p<0.05

**Statistical analysis:** The obtained data were coded, entered and processed on a personal computer using Statistical Package of Social Science (SPSS) for analysis of the results. Level of significance selected for this study was 95% (p<0.05), (a confidence level of 95%). Tests of significance used in this study included: unpaired Student´s t -test for continuous data, Chi-square test for categorical data, Fisher´s exact test was carried out when the cells expected counts of <5, Multiple logistic regression was used to identify predictors of burnout. Data was presented in tables and graphs according to the type of variables.

## Results

The study included 31 family physicians and 140 hospital practitioners under 4 main categories. There was a statistical significant difference between family physicians and hospital physicians regarding gender as more than (80%) of family physicians were females compared to less than forty percent of hospital physicians (36.4%) were females. The workload was higher among hospital physicians as half of them (50%) used to consult more than 20 patients/day, while more than one third of the family physicians (38.7%) used to consult 10-20 patients/day.

Hospital physicians were more liable to burnout than family physicians as shown in Table 1 where that the percentage of hospital physicians (53.9%) who met study criteria for burnout (A “high” score on at least 2 of the three dimensions of MBI) was higher than family physicians who met the same criteria (41.94%) with a statistical significant difference (p=0.00)

**Figure 1:**
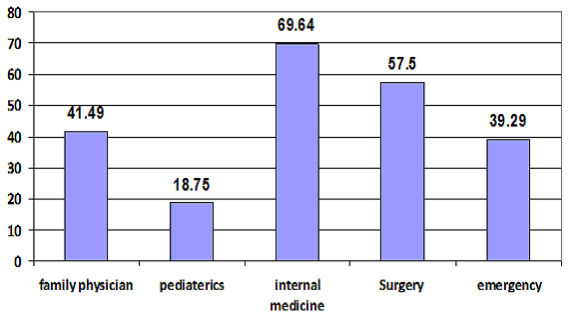
Percentage of burnout among the studied physicians in different specialties

Figure 1 represents that among study participants; the highest prevalence of burnout was found among Internal Medicine physicians (69.64%) followed by Surgeons (56.50%) and Family physicians (41.49%). Senior physicians suffered from burn out in a higher rates when compared to their juniors, as 63.6% of the senior physicians suffered from burnout compared to 42.6% of the juniors with a statistical significant difference between the two groups (p=0.006)

Table 2 represents the relationship between burnout and sociodemographic characteristics of the studied physicians. There was a statistical significant relationship between burnout and both of marital status and qualification of the studied physician as burnout was higher in married physicians (59.6%) than singles ones (39.7%), also the prevalence of burnout was higher in assistant lecturers (63.3%) compared to (42.6%) in residents/demonstrators. There is an inverse statistical significant relationship between burnout and both of practicing exercise and smoking in the studied physicians as the prevalence of burnout was lower in physicians practicing exercise (22%) compared to (61.5%) in those who did not practice exercise while burnout was more prevalent in non-smokers (60.1%) compared to (18.2%) in smokers.

Table 3 demonstrates the predictors of burnout from socio-demographic and work characteristics in the studied physicians. The risk of developing burnout increases to nearly eleven times more among hospital physicians compared to family physicians; married physicians have five times more risk compared to their single counterparts. While practicing exercise and being equal to or over 30 years of age are considered protecting factors from developing burnout (Table 4).

**Table 3:** Relationship between burnout and sociodemographic characteristics of the studied physicians

Sociodemographic characteristics	Burnout					Significance test (p-value)
	NO (*n*=82)		YES (*n*=89)		Total	
	No.	%	No.	%		
**Age group**						
**<30**	66	50.4	65	49.6	131	Fisher's exact (0.054)
**30-**	16	47.1	15	44.1	34	
**=35**	0	0.0	6	100.0	6	
**Gender**						
**Male**	32	42.1	44	57.9	76	X^2^=1.87 (0.171)
**Female**	50	52.6	45	47.4	95	
**Marital status**						
**Married**	40	40.4	59	59.6	99	Fisher's exact (0.007)*
**widow /Divorced**	1	100.0	3	100.0	3	
**Single**	41	60.3	27	39.7	68	
**Number of children**							
**1-2**	35	43.8	45	56.3	80	Fisher's exact (0.092)
**3-5**	3	100.0	0	0.0	3	
**Smoking**						
**Yes**	27	81.8	6	18.2	33	X^2^=18.79 (<0.001)*
**No**	55	39.9	83	60.1	138	
**Highest level of education**						
**Bachelor's degree**	54	57.4	40	42.6	94	X^2^=7.54 (0.006)*
**Master degree**	28	36.4	49	63.6	77	
**Practice exercise**						
**Yes**	32	78.0	9	22.0	41	X^2^=19.57 (<0.001)*
**No**	50	38.5	80	61.5	130	
**Having definite chronic diseases**						
**Yes**	4	66.7	2	33.3	6	Fisher's exact (0.428)
**No**	78	47.3	87	52.7	165	
**Having perceived financial problem**						
**Yes**	10	35.7	18	64.3	28	X^2^=2.01 (0.156)
**No**	72	50.3	71	49.7	143	
**Having social problem**						
**Yes**	28	56.0	22	44.0	50	X^2^=1.83 (0.176)
**No**	54	44.6	67	55.4	121	

* Statistically significant at p<0.05

**Table 4:** Multiple logistic regression analysis for predictors of burnout among studied physicians

Predictors	Coefficient	SE	p-value	Adjusted OR	95% CI	
**Hospital doctors**	2.394	0.839	0.004*	10.96	2.119	56.703
**Assistant Lecturers**	1.804	0.986	0.067	6.071	0.878	41.968
**Number of patients**	1.340	0.621	0.031*	3.818	1.129	12.905
**Married**	1.709	0.867	0.049*	5.524	1.011	30.194
**Smoker**	-1.844	0.957	0.054	0.158	0.024	1.032
**Practice exercise**	-3.872	0.901	<0.001*	0.021	0.004	0.122
**Age ≥ 30 years**	-2.767	1.140	0.015*	0.063	0.007	0.587
**Constant**	-1.208	1.151	0.294	0.299		

* Statistically significant at p < 0.05

**Reference categories**: Family physicians; Residents; Number of patients >10; Unmarried; non-smoker; No exercise and age <30 years

## Discussion

Burnout syndrome is characterized by loosing enthusiasm for work (emotional exhaustion), treating people as if they were objects (depersonalization), and having a sense that work is no longer meaningful (low personal accomplishment). The condition is as well associated with negative physiological, cognitive, psychological and behavioral manifestations. However, burnout syndrome is not a sign of weakness, mental illness or inability to cope with life therefore; burnout can be treated, overcome, and prevented [1].

This study was carried out to determine the prevalence of burnout and its related risk factors from sociodemographic and work characteristics in physicians working in Suez Canal University Hospitals. A total of 171 participants were evaluated for the presence of burnout and its associated risk factors using Maslach Burnout Inventory (MBI) and according to the three primary dimensions of burnout: emotional exhaustion, depersonalization and low personal accomplishment, the score was divided into low, moderate and high.

The current study showed that the prevalence of burnout in hospital physicians was higher than family physicians (53.9% versus 41.94%) with statistical significant difference between the two groups (p=0.001). Among hospital physicians the highest prevalence was found among the Internal Medicine physicians (69.64%). This study revealed a higher prevalence rate of burnout among study participants than what was found by others in the region; in Yemen it was (11.7%) [5] Whereas among Tunisia primary care doctors it was (33%) [6], and in Saudi Arabia the prevalence rate of burnout syndrome among female physicians working in the ministry of health hospitals in Jeddah city was (7.3 %) [7]. In other areas of the world several studies also showed that burnout is less prevalent than our study; Linzer et al (2001) and Bergner (2004), estimated that (22%) of physicians in the USA, (27%) of physicians in Great Britain [17], and (20%) of physicians in Germany suffer from burnout [18]. In another study conducted by Linzer et al (2009) involving 422 United States family practitioners and general internists in ambulatory clinics noted that (26.5%) reported burnout [19]. In Switzerland, Goebring et al. (2005) added a new aspect by looking at psychosocial and professional characteristics of burnout among 1755 Swiss primary care practitioners and the results showed that (32%) of the physicians had a high score on either the emotional exhaustion or the depersonalization subscales and were considered as having a moderate degree of burnout [20]. The differences in burnout rates across countries can be accounted by the job-demands-resources model which conceptualizes burnout as a consequence of the imbalance between job pressure and available resources. Healthcare professionals working in areas known for the low burnout rates have lower occupational pressure and more resources, also the difference in economic and political circumstances among countries may play a major role. The previous mentioned view was emphasized by the results of other studies [21–23]

Regarding the risk factors of burnout among physicians, The results of this study revealed that being a hospital physician gives eleven times more risk to develop burnout than being a family physicians (OR=10.96), the result is expected due to the more complex, stressful and irregular pattern of life led by hospital physicians, as described by (Balch et al. 2009) [24]. The physician has to work for long hours dealing with life-and-death situations, carry out a high volume of procedures and contract with multiple simultaneous deadlines without complaining along with keeping emotions or personal problems from interfering with work. Regarding to burnout distribution according to qualification, the study results showed that prevalence of burnout among assistant lecturers (63.6%) was significantly higher than it in the residents/demonstrators (42.6%). This result came opposite to other studies that concluded individuals relatively new to their jobs scored higher on measures of burnout [25, 26]. On the other hand Gaines and Jermier (1983) did not find any differences in the prevalence of burnout according to length of work experience [27]

As for the sociodemographic variables, being 30 or older is considered as a weak protective factor against developing burnout (OR=0.063), this result comes consistent to another studies [28, 29]. According to the study findings, there was no statistical significant difference between male and female participants in the prevalence of burnout. This result comes in parallel to studies concluded that gender does not influence burnout development like that previously done in (SCUH) [30], and the survey that was conducted in the Netherlands on 1426 physicians in primary care and specialties in which the authors found no significant sex difference in burnout rates [19]. When observing the findings of the Physician Work life Study, a disagreement with this study results was found. The nationally representative random stratified sample of nearly 6000 physicians in primary care specialty which assessed burnout among US physician concluded that female physicians were (60%) more likely than male physicians to report signs or symptoms of burnout [31]

The current study showed that women tend to report higher depersonalization scores while men tend to report higher emotional exhaustion and low personal accomplishment scores. This result is coming inconsistent to the results of multiple studies [32]. This may be attributed to the difference of how female physicians in our culture try to accommodate to workload in addition to management of family responsibilities.

In the present study, there was a statistical significant relationship between burnout and marital status especially in low personal accomplishment domain, the prevalence of burnout is higher in married physicians (59.6%) than in singles ones (39.7%) and the risk of developing burnout is five times more among married women. This result is different from studies that found no differences in burnout scores based on the marital status of participants [33], and that considered marital status as protective factor from burnout [34]. This might be explained on the light of the limited time available for married female physicians to spend at work; Physicians appear to get their sense of personal accomplishment from the number of hours they work. Such an attitude can reflect the way in which physicians are being evaluated and the major aim they seek.

The current study findings showed presence of inverse statistical significant relationship between prevalence of burnout and exercise also exercise was found as a protective factor against burnout (OR=0.021). This relationship was expected from literature as it is known that one of the strategies proposed to prevent burnout is physical activity which is a method used to diminish daily stress and increase psychological well-being. Several studies have shown that. In some populations physical exercise correlates with small to moderate decreases in depression [35]. Exercise can reduce anxiety and improve psychological well-being [36]. Depending on the facts of burnout being related to depression, anxiety, and psychological well-being and that physical activity has a positive influence on these factors, these results can be explained.

The results of the current study showed that non smokers were at risk to develop burnout more than smokers. The explanation of this relationship can be obtained from (Cleveland Clinic, 2008) that mentioned, stressors have two categories: one resides outside the person including economic pressures, difficult work environments, and interpersonal conflicts; and the second resides within the person including personality patterns, patterns of thinking and acting, unrealistic expectations, unmet needs, and genetics. Individuals, who face stress, also feel emotional exhaustion besides other stress symptoms and they seek help by smoking to cope with stress [37]. On scientific base, such finding could be explained as cigarettes contain nicotine which is an important element for mood altering. Tobacco users feel satisfaction and they possess a false sensation of escaping from stress; in fact, smoking is actually a cruel illusion. Smoking also may enhance the tendency for raised blood pressure, muscle stretching, blood vessels constricting and less availability of oxygen to the brain and body [37]

Workload was one of the most studied occupational factors in relation to burnout defined either as quantitative demands (number of working hours or number of attended patients) or as perceived workload. The results of the current study showed presence of statistical significant relationship between number of patients seen by the physician per day and burnout as the percent of burnout increased from (43.8%) to (76.7%) with the decrease in number of consulted patients to less than ten per day. This result comes in agreement with the result of large number of studies which consider subjective job experience as a strong burnout antecedent [38–39] and coming opposite to other studies which considered number of patients per day a strong predictor for all burnout dimensions [40]. The reason of the current study result can be clarified from the conclusion of Mara B et al. (2012). “In burnout development perceived workload is the main determinant, while case load and work times contribute indirectly to burnout, through perceived workload” [41]

The present study reported that there was no statistical significant relationship between burnout and work hours/week which come in controversy with the literature that had systematically linked workload to burnout [38]. In the same time many studies highlighted that extended work shifts expose medical professionals to burnout and serious medical errors [42]. In a desire to reduce medical errors, the Accreditation Council for Graduate Medical Education (ACGME, 2003) limited the working hours for American junior doctors to 80 hours a week [43]. Studies confirm the positive impact of these regulations. Residents were more likely to be involved in serious medical errors when they worked 24-hour shifts while the number of errors was reduced (36%) under the new regulations [44]. Although the authors believe the results regarding the association between burnout and work-induced stress should be viewed cautiously and should be used primarily to generate hypotheses for future research, the authors doubt that these findings solely reflect biased reporting. Finally, this study is limited by its cross-sectional design. Future longitudinal studies are required to evaluate the possibility of a causal relationship between work-induced stress and burnout.

## Conclusion

There was a considerable prevalence of burnout among physicians working in clinical departments of (SCUH) and in family physicians working in family practice centers affiliated to (FM/SCU). Practicing exercise is one of the cheap and effective methods to reduce burnout. Smoking is an illusion that gives a false sensation of escaping stress. Perceived work load has significant relation to burnout. Being a hospital physicians or married are considered strong predictors for the occurrence of burnout.
